# Retrospective Analysis of Nosocomial SARS-CoV-2 Infections in Orthopedic and Traumatological Inpatients

**DOI:** 10.3390/healthcare11202765

**Published:** 2023-10-19

**Authors:** Lukas Dankl, Ulrich Crepaz-Eger, Rohit Arora, Friedemann Schneider

**Affiliations:** Department of Orthopaedics and Traumatology, Medical University of Innsbruck, Anichstraße 35, 6020 Innsbruck, Austria; lukas.dankl@i-med.ac.at (L.D.);

**Keywords:** SARS-CoV-2, COVID-19, nosocomial infection, orthopedics, traumatology

## Abstract

SARS-CoV-2 has had a measurable impact on the field of orthopedic and traumatological surgery. To date, scarce data on intramural SARS-CoV-2 infections in orthopedic and traumatological patients have been reported. Therefore, the aim of our study was to investigate the effect of nosocomial SARS-CoV-2 infections in orthopedic and traumatological inpatients regarding symptoms of infection, mortality, duration of hospitalization, and other relevant patient-dependent factors. Patients admitted to hospital for an orthopedic or traumatological indication were screened retrospectively for nosocomial SARS-CoV-2 infections and included in this study. An age-, sex-, and ICD 10-matched control group was assigned and demographic data, clinical symptoms of a SARS-CoV-2 infection as well as mortality, length of hospital stays, time to surgery, pre-existing conditions, LKF-points representing the financial effort, and the Charlson Comorbidity Index were collected. A significantly higher length of stay was observed in the SARS-CoV-2 group (25 days; 4–60; SD 12.5) when compared to the control group (11 days; 2–36; SD 7; *p* < 0.05). LKF points were significantly higher in the SARS-CoV-2 group (13,939 points vs. 8542 points). No significant difference in mortality could be observed. An infection with SARS-CoV-2 in inpatients significantly increases length of hospital stay and cost of treatment. Although no significant difference in mortality was found, care should be taken to avoid intramural SARS-CoV-2 infections, resulting in prolonged hospitalization, higher costs, and potentially further individual risks.

## 1. Introduction

Since the beginning of the pandemic in December 2019 [1], SARS CoV-2 has been a steady companion in clinical practice. To our current knowledge, SARS CoV-2 is primarily spread via respiratory droplets in close face-to-face contact and can be spread via asymptomatic, presymptomatic, and symptomatic carriers [2]. The meta-analysis of early patient-data showed a vast variety of symptoms related to a SARS CoV-2 infection, for example fever and fatigue, cough, dyspnea, muscle soreness, and leukopenia, with a fatality rate of 5.5% [3]. Asymptomatic infections seem to appear more commonly in young patients. The reported rates of asymptomatic infections differ widely in the literature [4]. SARS-CoV-2 also had a major impact on elective orthopedic surgery. While nearly all total joint arthroplasties were being canceled [5], physical rehabilitation and postoperative follow-ups were also affected by the pandemic [6,7]. In traumatology, a reduction in visits to the trauma emergency department was observed [8,9], while a relative increase in severity of injuries occurred [9,10].

Mortality seems to be higher in male patients and patients with older age [11]. This might partially be due to a rise in pro-inflammatory cytokines, which is observed in elderly patients with SARAS-CoV-2 infections [12]. Nevertheless, little is known about SARS-CoV-2 infections in trauma patients. Mi et al. [13] described the characteristics of ten fracture patients with SARS-CoV-2 infections. While the range of symptoms did not change, the clinical characteristics seemed to be more severe than for patients without fractures. Edwards et al. [14] also reported on ten trauma patients with concomitant SARS-CoV-2 infections. Three patients required preoperative ventilation and five patients required postoperative ventilation. One patient required ventilation after postoperative day eight and tested positive for SARS-CoV-2 at postoperative day 14.

A variety of different measures were taken to resume elective and acute surgery [15,16]. The European Society of Sports Traumatology, Knee Surgery and Arthroscopy has released guidelines to ensure the safest environment possible for resuming elective surgery [17]. They recommend the hospital stay be as short as possible. A thorough preselection of patients should be performed. Symptoms of a SARS-CoV-2 infection should be inquired about, and a PCR-test should be performed before admission. Personal protective equipment should be used appropriately.

Even if all recommendations are implemented, there is still a chance for SARS-CoV-2 transmission in the hospital. This is especially true in trauma patients, where, e.g., hand-hygiene might be difficult due to an injury [18]. Additionally, healthcare workers might also be a source for nosocomial SARS-CoV-2 infections. Rivett et al. [19] tested 1032 asymptomatic healthcare workers for SARS-CoV-2 and observed positive test results in 3% of cases. This might lead to uncontrolled staff-to-staff and staff-to-patient transmission. When swabs were taken in hospital rooms, viral RNA was detected on surfaces, in air samples, and in stool samples, which could also lead to nosocomial infections [20]. Additionally, undetected SARS-CoV-2 infections in patients can cause transmission to a substantial amount of hospital staff [21].

Lately, reports on healthcare- and hospital-acquired SARS-CoV-2 infections have started to appear in the literature [22,23]. While we observed several nosocomial SARS-CoV-2 infections in our department, only little information has yet been published on nosocomial SARS-CoV-2 infections in orthopedic and traumatological inpatients. Therefore, the aim of this study is to retrospectively analyze inpatient, who were tested negative at the time of admission, but contracted SARS-CoV-2 during their hospitalizations at our Department of Orthopedics and Traumatology regarding symptoms of infection, mortality, duration of hospitalization, and other relevant patient-dependent factors.

We hypothesized that the nosocomial contraction of a SARS-CoV-2 infection during the hospital stay could lead to a prolonged hospitalization for trauma patients as well as increased mortality compared to non-infected patients.

## 2. Materials and Methods

Patients who entered hospital care for a traumatological or orthopedical indication from 1 January 2020 to 1 May 2021 were screened retrospectively and were included in this study if they met the following inclusion criteria.

- Orthopedical or traumatological main diagnosis.
- Negative SARS-CoV-2 PCR test result at the time of admission.
- At least one positive SARS-CoV-2 PCR test result during the time of hospitalization.

A SARS-CoV-2 PCR test was performed routinely as part of contact tracing or if the patient showed symptoms of a SARS-CoV-2 infection. These patients were included in the “SARS-CoV-2 group”.

Patients were excluded if they had a history of a previous SARS-CoV-2 infection, had a positive SARS-CoV-2 PCR test result at the time of admission, or were treated for a non-orthopedical or traumatological diagnosis.

To compare the outcome parameters, an age-, sex-, and ICD-10-matched “control group” was assigned. Patients were excluded if they had a history of a previous SARS-CoV-2 infection, had a positive SARS-CoV-2 test result at the time of admission, or developed a SARS-CoV-2 infection during hospitalization. If an individual patient in the SARS-CoV-2 group could not be matched to a patient hospitalized during the time of observation, our patient data were screened further back into the past until a matching patient was found and included (period of time not exceeding 10 years).

In both groups, the demographic data and clinical symptoms of a SARS-CoV-2 infection, as well as mortality, length of hospital stays (LOS), time to surgery, pre-existing conditions, and the Charlson Comorbidity Index were collected. 

To compare the financial effort in the two groups, LKF points for the individual stay were calculated. The LKF system, which is used in Austria, allows for the billing of an inpatient stay at a hospital. Points are calculated based on so-called “achievement-oriented diagnosis case groups” in combination with other factors, such as individual medical interventions, patient age, effort in patient care, and the infrastructure of the ward [24].

Data were collected in an Excel sheet (Excel 365, Version 2111, Microsoft Corporation, Redmond, WA, USA). Statistical analysis was carried out with IBM SPSS (IBM SPSS Statistics, Version 24.0.0.1, IBM Corporation, Armonk, NY, USA). For all numeric data, mean, standard deviation, range, and 95% confidence interval were calculated. For categorical data, frequencies and percentages were calculated. To test for normal distribution, a one-sample Kolmogorov–Smirnov test was used. A comparison of the SARS-CoV-2 group and the control group was performed. For numeric parameters, an independent sample *t*-test or a Mann–Whitney U-test was used depending on the normal distribution. For categorical data, frequencies and percentages were compared to the estimated values using a chi-square test. Mean, range and standard deviation (SD) were stated for all values. Significance level was set to *p* < 0.05.

## 3. Results

During the time of observation, 27 patients met the inclusion criteria for this study. Therefore, we recruited 27 patients for the control group. There was no significant difference in the demographic data between the SARS-CoV-2 group and the control group (Table 1). In the SARS-CoV-2 group, the mean time to positivity was 11 days (3–44; SD 8.9). Reasons for admission are listed in Table 2.

We observed a significantly higher number of days spent in hospital in the SARS-CoV-2 group (25 days; 4–60; SD 12.5) when compared to the control group (11 days; 2–36; SD 7; *p* < 0.05). 

An operation was performed on 23 of the 27 patients in the SARS-CoV-2 group as well as the control group. There was no significant difference in mean time to surgery in the SARS-CoV-2 group (2 days, 0–6; SD 2.1) when compared to the control group (2 days, 0–7; SD 2, *p* = 0.48).

Symptoms and clinical presentation of the patients is summarized in Table 3. No significant difference in mortality could be observed between the two groups.

**Figure 1:**
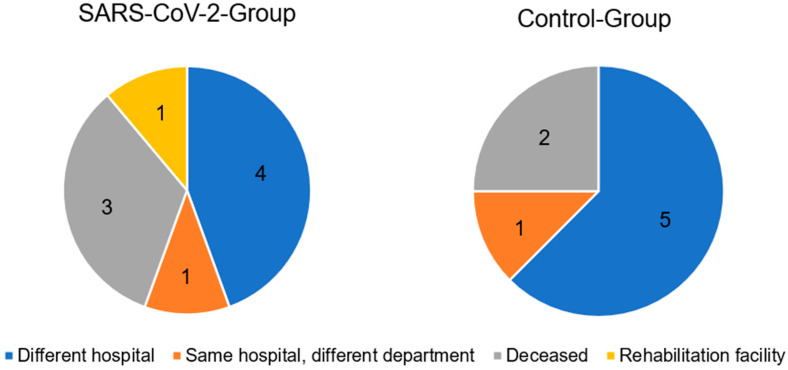
Distribution of patients who could not be discharged home.

In the SARS-CoV-2 group, 18 patients were discharged home. In the control group, 19 patients were discharged home. The distribution of the remaining patients can be seen in Figure 1.

LKF points were significantly higher in the SARS-CoV-2 group (*p* < 0.05) with a mean of 13,939 points (5022–46,077; SD 9377) in the SARS-CoV-2 group and 8542 points (2086–28,767; SD 5973) in the control group.

When performing Pearson’s correlation analysis in the SARS-CoV-2 group, we observed significantly moderate to strong correlations between age and the Charlson Comorbidity Index (r = 0.637, *p* < 0.05); time to positivity and LOS (r = 0.734, *p* < 0.05); as well as the Charlson Comorbidity Index and number of pre-existing conditions (r = 0.735, *p* < 0.05). Moderate correlations were observed between LOS and number of LKF points (r = 0.574, *p* < 0.05); LOS and Charlson Comorbidity Index (r = 0.541, *p* < 0.05); age and number of pre-existing conditions (r 0.521, *p* < 0.05); time to positivity and number of preexisting conditions (r = 0.466, *p* < 0.05), as well as the Charlson Comorbidity Index (r = 0.560, *p* < 0.05) and number of LKF points (r = 0.475, *p* < 0.05); time to surgery and number of LKF points (r = 0.588, *p* < 0.05); CRP and number of LKF points (r = 0.465, *p* < 0.05), as well as the Charlson Comorbidity Index and number of LKF points (r = 0.460, *p* < 0.05). Correlation was weak but significant for age and CRP (r = 0.384, *p* < 0.05) as well as LOS (r = 0.396, *p* < 0.05).

In the control group, a medium to strong correlation was observed between age and Charlson Comorbidity Index (r = 0.783, *p* < 0.05) as well as number of pre-existing conditions (r = 0.515, *p* < 0.05); number of pre-existing conditions and the Charlson Comorbidity Index (r = 0.715, *p* < 0.05); and LOS and number of LKF points (r = 0.628, *p* < 0.05). A weak but significant negative correlation was observed between count of leucocytes and the Charlson Comorbidity Index (r = −0.396, *p* < 0.05).

## 4. Discussion

The most important finding of this study is that an infection with SARS-CoV-2 during the inpatient stay significantly increases the time of hospital stay and the financial effort, leading to increased costs for the hospital operator. 

The LOS was more than doubled if a nosocomial infection with SARS-CoV-2 occurred. When comparing the observed LOS with data in the literature, the 11 days for the control group matches the reported values well. Brasel et al. observed a mean LOS of 9.6 days [25], and Moore et al. reported an overall mean LOS of 8.6 days with 9.6 days in the age group of 65–74 years (which matches the mean age of the control group) [26]. Brotemarkle et al. reports a mean LOS of only 4.3 days. In contrast to our study, 57.6% of patients were discharged to a facility for further care [27], while in our sample, only 22.2% were discharged to a rehabilitation facility or another hospital. 

These findings are in line with results published in the literature. As a part of the COPE-Nosocomial study, Carter et al. analyzed data of 1564 patients from 10 hospitals. They observed nosocomial infections in only 12.5% of all SARS-CoV-2 cases. A nosocomial SARS-CoV-2 infection increased LOS, but there was no difference in seven-day mortality compared to patients with a community acquired SARS-CoV-2 infection [28]. 

Increase in LOS in the SARS-CoV-2 group might have different reasons. Although more than half of the patients stayed asymptomatic, they had to be quarantined. In Austria, the state-mandatory quarantine was 14 days during the observation period. Patients who underwent surgery needed special medical care, which could not be guaranteed during quarantine if the patients were discharged home. In cases of overall symptoms and oxygen-dependency, a discharge could only be approved if the patient’s condition allowed for it.

LKF points were increased by 61.3% in the SARS-CoV-2 group. This is likely due to the increased LOS, which, among other factors, is used to calculate LKF points. We were also able to observe a significant positive correlation between LKF points and CRP levels as well as the Charlson Comorbidity Index. This might be a representation of the “individual medical interventions”, which also influences the calculation of LKF points. This correlation between the Charlson Comorbidity Index and LOS; however, this was not present in the control group. A patient with a higher Charlson Comorbidity Index might suffer more severely from a SARS-CoV-2 infection and might therefore need more, if not intensive care to recover. Days on an intensive care unit and increased intensity of medical interventions have a strong influence on LKF points [24]. 

Clinical symptoms of an acute SARS-CoV-2 infection were only present in 51.8% of patients in the SARS-CoV-2 group. This corresponds well with the current literature, which reports rates of asymptomatic infections ranging from 32% [2] up to 51.7% [29]. In our sample group, 12 of the 14 patients experiencing specific symptoms were dependent on external oxygen supply. According to the clinical characteristics of a COVID-19 infection, this would classify as a severe infection. While fever and leukopenia were more common in the SARS-CoV-2 group, there was no significant difference in CRP values. As an acute phase protein, CRP is elevated in trauma as well as infection and inflammation [30]. Therefore, it was not surprising to find no differences between the two groups in this regard.

There was no significant difference in mortality in the two groups. In the SARS-CoV-2 group, three patients died while in the control group, two patients died during hospital care. All these patients were over the age of 70 and had at least four relevant pre-existing conditions and a Charlson Comorbidity Index of five. Giannoudis et al. showed that the mortality in trauma patients increases significantly with older age and is almost 50% in patients over the age of 75 [31]. In patients with isolated femoral neck fractures, Major et al. found an in-hospital mortality of 7.5% [32]. Additionally, a Charlson Comorbidity Index of five indicates a ten-year survival of 21% [33]. Because of the inherently high mortality in elderly trauma patients, our sample size might have been too small to detect a significant difference in mortality between the two groups.

Lakhani et al. evaluated data from 19 patients who acquired a nosocomial SARS-CoV-2 infection in an orthopedic surgery department. While the authors observed a significant increase in LOS and mortality in patients with a nosocomial SARS-CoV-2 infection, they compared their findings to non-infected patients admitted at the same observation period. They reported significant differences in critical patient characteristics such as age, gender, and residence [34]. A strength of our study is the implementation of an ICD-10-, age-, and sex-matched control group which allows for the direct comparison of outcomes. Oputa et al. also showed a significantly increased 30-day and 120-day mortality for patients with hip fractures with a concurrent SARS-CoV-2 infection [35]. 

One limitation of this study is the small sample size of only 27 infections with SARS-CoV-2 in inpatients at our department due to the overall low in-hospital infection rate. While this is generally a good thing, it results in a rather small total population of only 54 individuals. This sample size may be too small to detect minor differences in mortality. During the observation period, FFP-2 masks were mandatory and the hospital staff was required to have regular COVID-19 swabs. Furthermore, personal protective equipment was provided to minimize patient and staff infection. 

In addition, it must be noted that in this study cohort, more female patients were included. The sex composition of the observed study cohort was applied to the control group accordingly to minimize risk of bias. Nonetheless, as male sex seems to be a risk factor for a more severe infection [11], the severity of symptoms might be underestimated in this study.

This study was performed retrospectively. Therefore, data on differences in functional outcome and long-term survival cannot be reported. Paths of infection were not reported because they were not traceable in all patients. While some patients had contact with other SARS-CoV-2 positive patients or infected staff, the course of infection remains unclear in most of the patients.

## 5. Conclusions

An infection with SARS-CoV-2 in inpatients hospitalized for a traumatological or orthopedical indication significantly increases length of hospital stay and cost of treatment. We could not observe a significant difference in mortality between the two groups. Care should be taken to avoid intramural SARS-CoV-2 infections.

## Figures and Tables

**Table 1:** Demographic data of the SARS-CoV-2 and the control group.

	SARS-CoV-2	Control
Age	70.7 y (27–90; SD 14,6)	69 y (21–90; SD 16.6)
Sex	8 male; 19 female	8 male; 19 female

**Table 2:** Diagnoses at admission.

Reason for Admission	Number (n=)
Fracture of the lower leg and foot	6
Fracture of the proximal femur	5
Arthrosis of hip or knee	4
Fracture of the humeral head	3
Joint infection	2
Craniocerebral injury	2
Complication after implantation of an endoprosthesis	2
Degenerative spine conditions	1
Fracture of the pelvis	1
Vertebral fracture	1

**Table 3:** Symptoms and clinical presentation of the patients.

	SARS-CoV-2 Group	Control Group	*p*-Value
CRP elevated (n)	24 (88.9%)	23 (85.2%)	0.64
CRP normal (n)	3 (11.1%)	4 (14.8%)	
CRP (mg/dL)	6.42 (5.75; 0.14–24.09)	6.64 (7; 0.06–28.54)	0.71
Leucocytes decreased (n)	10 (37%)	2 (7.4%)	
Leucocytes elevated (n)	1 (3.7%)	10 (37%)	
Leucocytes normal (n)	16 (59.3%)	15 (55.6%)	<0.05
Leucocytes (109/L)	5.9 (4.4; 1.0–23.4)	9.2 (3.4; 2.6–16.2)	<0.05
Oxygen-dependency (n)	12 (44.4%)	2 (7.4%)	<0.05
Fever (n)	11 (40.7%)	2 (7.4%)	<0.05
COV-specific symptoms (n)	14 (51.9%)	0	-
COV-specific treatment (n)	14 (51.9%)	0	-
Deceased (n)	3 (11.1%)	2 (7.4%)	0.64

## Data Availability

Data is unavailable due to privacy or ethical restrictions.
